# Intrahost evolution leading to distinct lineages in the upper and lower respiratory tracts during SARS-CoV-2 prolonged infection

**DOI:** 10.1093/ve/veae073

**Published:** 2024-08-31

**Authors:** Majdouline El Moussaoui, Sebastien Bontems, Cecile Meex, Marie-Pierre Hayette, Marie Lejeune, Samuel L Hong, Simon Dellicour, Michel Moutschen, Nadine Cambisano, Nathalie Renotte, Vincent Bours, Gilles Darcis, Maria Artesi, Keith Durkin

**Affiliations:** Department of Infectious Diseases and General Internal Medicine, University Hospital of Liège, 1 Avenue de l’Hôpital, Liège 4000, Belgium; Department of Microbiology, University Hospital of Liège, 1 Avenue de l’Hôpital, Liège 4000, Belgium; Department of Microbiology, University Hospital of Liège, 1 Avenue de l’Hôpital, Liège 4000, Belgium; Department of Microbiology, University Hospital of Liège, 1 Avenue de l’Hôpital, Liège 4000, Belgium; Department of Hematology, University Hospital of Liège, 1 Avenue de l’Hôpital, Liège 4000, Belgium; Department of Microbiology, Immunology and Transplantation, Laboratory for Clinical and Epidemiological Virology, Rega Institute, KU Leuven, 49 Herestraat, Leuven 3000, Belgium; Department of Microbiology, Immunology and Transplantation, Laboratory for Clinical and Epidemiological Virology, Rega Institute, KU Leuven, 49 Herestraat, Leuven 3000, Belgium; Spatial Epidemiology Lab (SpELL), Université Libre de Bruxelles, 50 Avenue Franklin Roosevelt, Bruxelles 1050, Belgium; Department of Infectious Diseases and General Internal Medicine, University Hospital of Liège, 1 Avenue de l’Hôpital, Liège 4000, Belgium; Department of Human Genetics, University Hospital of Liège, 1 Avenue de l’Hôpital, Liège 4000, Belgium; Laboratory of Human Genetics, GIGA Institute, University of Liège, 1 Avenue de l’Hôpital, Liège 4000, Belgium; Department of Human Genetics, University Hospital of Liège, 1 Avenue de l’Hôpital, Liège 4000, Belgium; Laboratory of Human Genetics, GIGA Institute, University of Liège, 1 Avenue de l’Hôpital, Liège 4000, Belgium; Department of Human Genetics, University Hospital of Liège, 1 Avenue de l’Hôpital, Liège 4000, Belgium; Laboratory of Human Genetics, GIGA Institute, University of Liège, 1 Avenue de l’Hôpital, Liège 4000, Belgium; Department of Infectious Diseases and General Internal Medicine, University Hospital of Liège, 1 Avenue de l’Hôpital, Liège 4000, Belgium; Department of Human Genetics, University Hospital of Liège, 1 Avenue de l’Hôpital, Liège 4000, Belgium; Laboratory of Human Genetics, GIGA Institute, University of Liège, 1 Avenue de l’Hôpital, Liège 4000, Belgium; Department of Human Genetics, University Hospital of Liège, 1 Avenue de l’Hôpital, Liège 4000, Belgium; Laboratory of Human Genetics, GIGA Institute, University of Liège, 1 Avenue de l’Hôpital, Liège 4000, Belgium

**Keywords:** SARS-CoV-2, COVID-19, within-host evolution, genetic compartmentalization, intrahost evolution, chronic infection, prolonged infection, immunosuppression, ORF8

## Abstract

Accumulating evidence points to persistent severe acute respiratory syndrome coronavirus 2 (SARS-CoV-2) infections in immunocompromised individuals as a source of novel lineages. While intrahost evolution of the virus in chronically infected patients has previously been reported, existing knowledge is primarily based on samples from the nasopharynx. In this study, we investigate the intrahost evolution and genetic diversity that accumulated during a prolonged SARS-CoV-2 infection with the Omicron BF.7 sublineage, which is estimated to have persisted for >1 year in an immunosuppressed patient. Based on the sequencing of eight samples collected at six time points, we identified 87 intrahost single-nucleotide variants, 2 indels, and a 362-bp deletion. Our analysis revealed distinct viral genotypes in the nasopharyngeal (NP), endotracheal aspirate, and bronchoalveolar lavage samples. This suggests that NP samples may not offer a comprehensive representation of the overall intrahost viral diversity. Our findings not only demonstrate that the Omicron BF.7 sublineage can further diverge from its already exceptionally mutated state but also highlight that patients chronically infected with SARS-CoV-2 can develop genetically specific viral populations across distinct anatomic compartments. This provides novel insights into the intricate nature of viral diversity and evolution dynamics in persistent infections.

## Introduction

The severe acute respiratory syndrome coronavirus 2 (SARS-CoV-2) pandemic has been characterized by the repeated emergence of divergent lineages with a significant growth advantage over contemporary variants (Obermeyer et al. 2022, Markov et al. 2023). These variants of concern (VOCs) generally exhibit enhanced transmissibility and the ability to evade pre-existing acquired immunity, posing significant challenges to the containment of SARS-CoV-2 spread. The World Health Organization has designated five VOCs of SARS-CoV-2, namely, the Alpha (B.1.1.7), Beta (B.1.351), Gamma (P.1), Delta (B.1.617.2), and lastly Omicron (B.1.1.529) in November 2021 (Markov et al. 2023). Compared with previous variants, Omicron encompassed a substantially larger number of unique changes in the spike glycoprotein, contributing to antigenic distance and probably occurring at least partly in the context of a chronic infection. The emergence of Omicron raised immediate global concerns due to its rapid global spread, attributable to its remarkable ability to evade immune responses induced by prior vaccination or infections, increased transmission rate, and high tropism for nasal epithelial cells (Hui et al. 2022, Viana et al. 2022). Subsequently, several antigenically drifted sublineages of Omicron (e.g. BA.4/5, BA.2.75.2, BA.4.6, BQ.1.1, and XBB) emerged and supplanted prior subvariants (Roemer et al. 2023). Compared to the BA.2 lineage, BA.4 and BA.5 have demonstrated increased escape from antibody neutralization, attributed to mutations at antigenically potent receptor-binding domain (RBD) positions, notably, L452R and F486V (Pather et al. 2023). Although a large fraction of the mutations are localized in the spike’s RBD or N-terminal domain, accumulating evidence suggests that mutations impacting viral genes outside of spike play critical roles in the fitness of Omicron sublineages (Jung et al. 2022). More recently, the second half of 2023 saw the emergence of the novel lineage BA.2.86, an Omicron sublineage, which had accumulated over 30 amino acid changes in the spike protein compared to its BA.2 progenitor. Although BA.2.86 initially showed modest growth, the acquisition of additional mutations led to the daughter lineage JN.1, which subsequently became the dominant lineage worldwide (Kaku et al. 2024).

Newly emergent VOCs generally perch atop a long ancestral phylogenetic branch, rooted in a historic lineage absent from the current variant landscape (Roemer et al. 2023). While SARS-CoV-2 spillover/spillback to and from an animal host could potentially generate similar long phylogenetic branches (Feng et al. 2023), recent evidence suggests that immunocompromised individuals with persistent infections are the main source of these divergent lineages (Carabelli et al. 2023, Markov et al. 2023). Since the early stages of the pandemic, long-term replication has been documented in chronically infected immunocompromised individuals, accelerating intrahost viral evolution and leading to the acquisition of amino acid changes that often overlap with those identified in VOCs (Avanzato et al. 2020, Choi et al. 2020, Kemp et al. 2021). Therefore, monitoring of SARS-CoV-2 mutations and understanding intrahost viral evolution in immunocompromised individuals remain a priority.

In this report, we characterized the intrahost genetic diversity and evolution of SARS-CoV-2 in an immunocompromised patient with B-cell depletion due to diffuse large B-cell lymphoma (DLBCL), showing persistent SARS-CoV-2 Omicron BF.7 replication, suspected to have spanned over a year. In contrast to most chronic SARS-CoV-2 infections reported in the literature, we sequenced SARS-CoV-2 from different specimen sites collected on the same day, including nasopharyngeal (NP) swab, endotracheal aspirate (ETA), and bronchoalveolar lavage (BAL) samples. The resulting sequences revealed a deep divergence in the viral lineages at opposite ends of the respiratory tract, with a mixture of viral populations in the ETA sample. These findings highlight that NP samples only capture a subset of the diversity present in chronic infection and that a negative test does not rule out the presence of a SARS-CoV-2 reservoir in immunocompromised individuals.

## Results

### The identification of a persistent SARS-CoV-2 infection in an immunosuppressed host

The patient, in their 60s, suffered from follicular lymphoma (Grade 2, clinical stage IV), which subsequently progressed into DLBCL. During the end of 2021, they underwent treatment with four cycles of rituximab with cyclophosphamide, doxorubicin, vincristine, and prednisone (R-CHOP), followed by two cycles of rituximab alone, resulting in a complete remission of the disease. Since February 2022, the patient has been receiving bimonthly infusions of rituximab as maintenance therapy. In addition, the patient’s medical history was also notable for a first mild episode of Coronavirus disease 2019 (COVID-19) in January 2022, which resolved over a few days. In April, reverse transcription–polymerase chain reaction (RT-PCR) for SARS-CoV-2 on an NP sample was negative. Of note, the patient had received three doses of vaccine (two doses of ChAdOx1-S followed by one of BNT162b2) between March and October 2021, resulting in detectable antitrimeric spike protein-specific IgG antibodies in June 2021.

In July 2022, they were admitted for fever, cough, shortness of breath, haemoptysis, diarrhoea, and generalized weakness, evolving over a period of 4 days. Chest computed tomography (CT) showed diffuse and bilateral ground-glass opacities, with moderate involvement (20%–25%) taking consolidated appearance in the middle lobe, as well as a small bilateral pleural effusion. SARS-CoV-2 PCR performed on an NP swab sample was positive [RdRp gene, cycle threshold (*C*_t_) = 35], and the patient was diagnosed with COVID-19 pneumopathy. They were treated with a 5-day course of molnupiravir and required transient oxygen supplementation, but eventually evolved favourably and were discharged home 7 days after admission. The maintenance therapy with rituximab was also discontinued.

During the subsequent months, the patient reported intermittent fatigue, low-grade fever, cough, blood-tinged sputum, and dyspnoea. Chest CT revealed diffuse ground-glass opacities with new upper left and middle lobe consolidations suggestive of organizing pneumonia. After exclusion of infectious alternative diagnoses, including notably a negative RT-PCR for SARS-CoV-2 on an NP sample, treatment with methylprednisolone (8 mg/day) was initiated in September 2022, resulting in partial resolution of the symptomatology over a period of 3 weeks. However, shortly after discontinuing corticosteroids, symptoms recurred, prompting their reintroduction by the end of October 2022. Between December 2022 and April 2023, the clinical condition was marked by a systematic recurrence of respiratory symptoms following each attempt of corticosteroid discontinuation. Of note, RT-PCR for SARS-CoV-2 performed on a BAL sample remained negative in April 2023.

**Figure 1.**
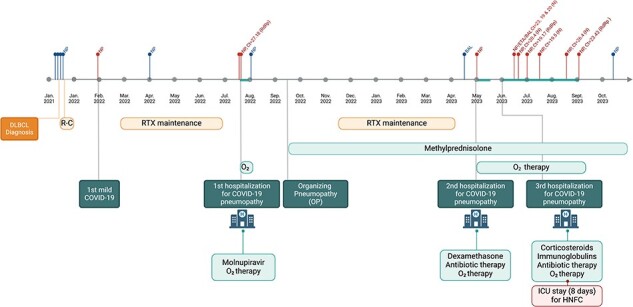
Timeline of the clinical history and disease course. The figure illustrates the timeline progression of the patient’s COVID-19 infection along with relevant clinical events. The top section displays the SARS-CoV-2 RT-PCR results and *C*_t_ values in NP, ETA, and BAL samples over time. Positive test results are indicated in red, while negative tests are represented in blue. The lower section provides a comprehensive overview of the patient’s clinical history including the history of DLBCL and its treatment, three hospitalizations (July 2022, May 2023, and from June 2023 to September 2023, including an 8-day ICU stay in June 2023) related to COVID-19, and the therapeutic interventions administered to the patient. Abbreviations: HFNC, high-flow nasal cannula therapy, R-C, R-CHOP; RTX, rituximab.

In May 2023, the patient was hospitalized for fever, cough, and moderate respiratory failure that required oxygen supplementation. Chest CT showed a marked increase in diffuse and bilateral ground-glass opacities with severe lung involvement (75%). SARS-CoV-2 RT-PCR on an NP swab sample was positive, confirming the diagnosis of COVID-19 pneumopathy. The patient was treated with dexamethasone and a 7-day course of antibiotic therapy due to a high suspicion of bacterial coinfection. Following an initial clinical amelioration, permitting discharge home with oxygen supplementation and oral corticosteroids, they were readmitted in June due to a subsequent exacerbation of respiratory insufficiency. This progression necessitated an 8-day stay in the intensive care unit (ICU) for the administration of high-flow nasal cannula therapy. After multiple rounds of antibiotic therapy for bacterial superinfections, intravenous immunoglobulin, and high-dose corticosteroid pulses, they gradually improved and were able to return home after ∼3 months of hospitalization. The persistence of the infection during the period was confirmed by positive RT-PCR tests on ETA, BAL, and multiple NP samples, with *C*_t_ values ranging from 16.1 to 26.4, between May 2023 and September 2023 (126 days). Finally, in October 2023, the RT-PCR test yielded a negative result. The clinical course of the infection is summarized in Fig. 1, while the evolution of imaging is displayed in Supplementary Fig. S1.

### Genomic sequencing points to chronic infection lasting over a year

**Figure 2.**
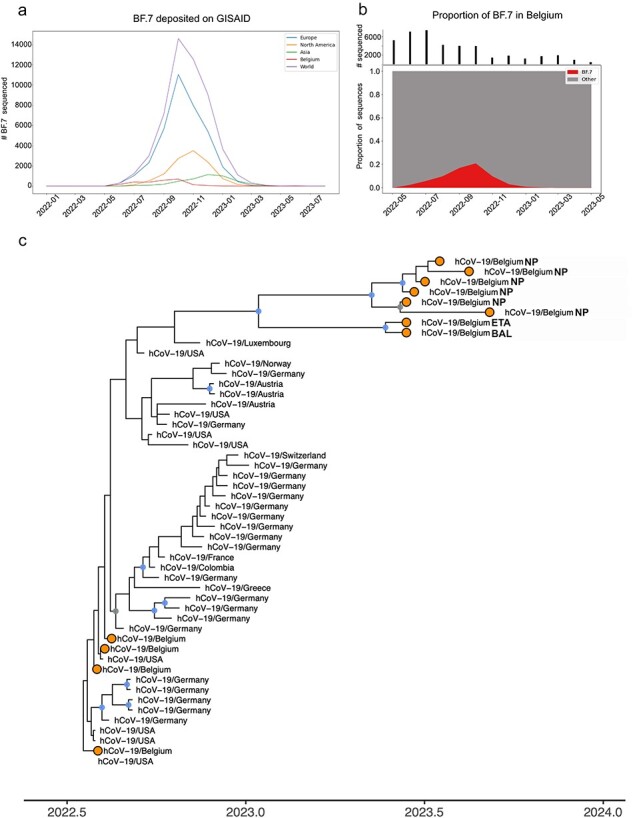
Dynamics of the BF.7 sublineage and phylogenetic tree. (a) Temporal distribution of BF.7 sublineage (BA.5.2.1.7) samples from GISAID. (b) Proportion of public Belgian genomes from GISAID belonging to the BF.7 sublineage sampled between May 2022 and May 2023. The number of samples that were sequenced each month is indicated earlier. (c) Maximum clade credibility tree resulting from a time-scaled phylogenetic inference carried out to display the evolutionary relationships among the eight SARS-CoV-2 BF.7 sequences from the patient and the most closely related genomes identified in the USHER global phylogenetic tree. Belgian genomes are marked in orange, and nodes with posterior probability values of >.7 and >.9 are given in grey and blue, respectively.

In total, we sequenced eight samples from the patient collected over an 85-day period, between June and September 2023. These samples were found to be derived from the almost extinct Omicron BA.5 sublineage BF.7 (Fig. 2a). This sublineage saw its Belgian peak in October 2022, accounting for 17.4% of sequenced samples (Fig. 2b). The sublineage declined in the following months, with the last Belgian example sequenced in February 2023. The patient’s consensus genomes were placed onto the existing SARS-CoV-2 phylogenetic tree using the Ultrafast Sample placement on Existing tRee (UShER) tool (Turakhia et al. 2021). They sat at the end of a long phylogenetic branch, separated by over 25 single-nucleotide variants (SNVs) from their closest relatives, which were collected towards the end of 2022 (Fig. 2c). A consensus genome representing the 72 SNVs/deletions fixed in the eight samples matched three BF.7 genomes (EPI_ISL_14483715, EPI_ISL_14483944, and EPI_ISL_15222264) collected between July and September 2022 (Supplementary Fig. S2 and Supplementary Table S1).

The patient tested positive for SARS-CoV-2 in January 2022 with a subsequent negative test in April. At this time, BF.7 was observed at very low frequencies worldwide (Fig. 2a) and was not detected in Belgium until May 2022 (Fig. 2b), making this first infection an unlikely time point for the establishment of a persistent infection. The patient again tested positive in July 2022, and at this time, BF.7 was the fifth most common lineage observed in Liège, representing 6.3% of samples sequenced during the week they tested positive (Supplementary Fig. S3). The patient had negative tests in August 2022 and in April 2023, but again tested positive in May 2023 and was consistently positive in subsequent tests (Fig. 1). Unfortunately, only samples from June 2023 forward were sequenced, and as a consequence, it is impossible to draw a definitive conclusion. Nevertheless, based on the viral lineage observed in the samples taken from June 2023 on, it would appear that the infection acquired in July 2022 never cleared (despite intervening negative PCR tests), which suggests that the patient was chronically infected for over a year.

### Distinct viral populations in the upper and lower respiratory tracts

While the presence of a long branch leading to the patient samples is expected in a prolonged infection, the relative placement of the consensus genomes was initially somewhat surprising.

**Figure 3.**
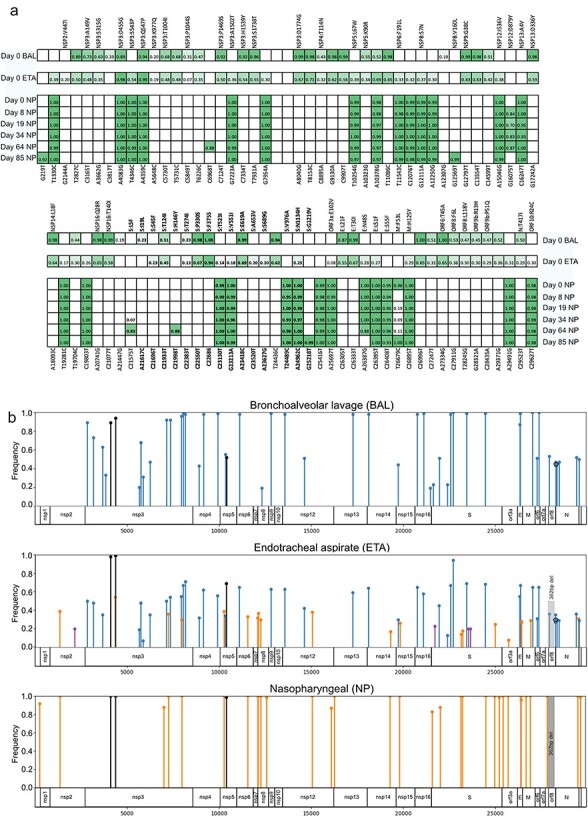
Evolution of patient-specific variants over time according to different anatomic compartments. (a) Frequency of iSNV across the upper and lower respiratory tracts over time, Day 0 corresponds to the first samples sequenced, taken in June 2023. The top panel shows iSNV at the 5' end of the virus; the bottom shows the 3' end. The genomic position corresponding to ancestral Wuhan-1 (MN908947.3) is shown on the bottom, while amino acid changes are indicated on top. Colour intensity reflects allele frequency. (b) Patient-specific mutations from upper and lower respiratory tracts are mapped to the SARS-CoV-2 genome, and viral proteins are shown at bottom. The graph shows the allelic frequency of variants found in the NP and BAL samples, and variants found in all six ETA samples are combined. The height of the line represents the allele frequency. In the NP graph, the highest allele frequency observed in the six time points is used. Variants that are exclusively found in BAL and ETA samples are represented in blue, those found only in NP and ETA are shown in orange, and ETA-exclusive variants are shown in purple. Variants that are present in all the samples are represented in black. iSNVs are represented by circles, indels by polygons, and deletions by shaded rectangles.

Notably, the six NP samples formed a cohesive cluster, whereas the BAL and ETA samples occupied a distinct branch that showed early divergence from the NP samples, indicating a long period of independent evolution. Across all samples, we identified 87 unique intrahost SNVs (iSNVs; Fig. 3a), 2 indels, and a 362-bp deletion (MN908947.3:27818–28180) involving the accessory protein-coding genes ORF7a and ORF8 (Fig. 3b; Supplementary Fig. S4 and Supplementary Table S2). All iSNVs had an allele frequency of 19% or more in at least one sample. Interestingly, substantial disparities were observed in genetic variants found in upper and lower respiratory tract samples. In the first NP sample taken in June 2023, we observed 29 iSNVs as well as the 362-bp deletion, all with an allele frequency of 95% or higher (Fig. 3a). In contrast, in the BAL sample collected on the same date, 50 iSNVs and 2 indels were found, with frequencies varying between 19% and 100% (Fig. 3a). Remarkably, only three of these genetic variants were found in both samples (Fig. 3b), explaining the significant divergence observed in the branches of the phylogenetic tree (Fig. 2c). Regarding the ETA sample, we observed 71 iSNVs with allele frequencies ranging from 7% to 99%. Most of the iSNVs found in ETA samples were also identified in NP or BAL samples. Only four iSNVs were unique to the ETA sample (Fig. 3b). In comparison to the BAL and ETA samples, iSNVs identified in NP samples generally showed high allele frequencies, with variants appearing and disappearing relatively abruptly between samples (Fig. 3a). Over an 85-day period, we observed the emergence of six iSNVs and one reversion in the subsequent five NP samples. Notably, in the last sample, only three of the new iSNVs and the reversion persisted, as three had been lost in the intervening time (Fig. 3a).

### iSNV mutational patterns

**Figure 4.**
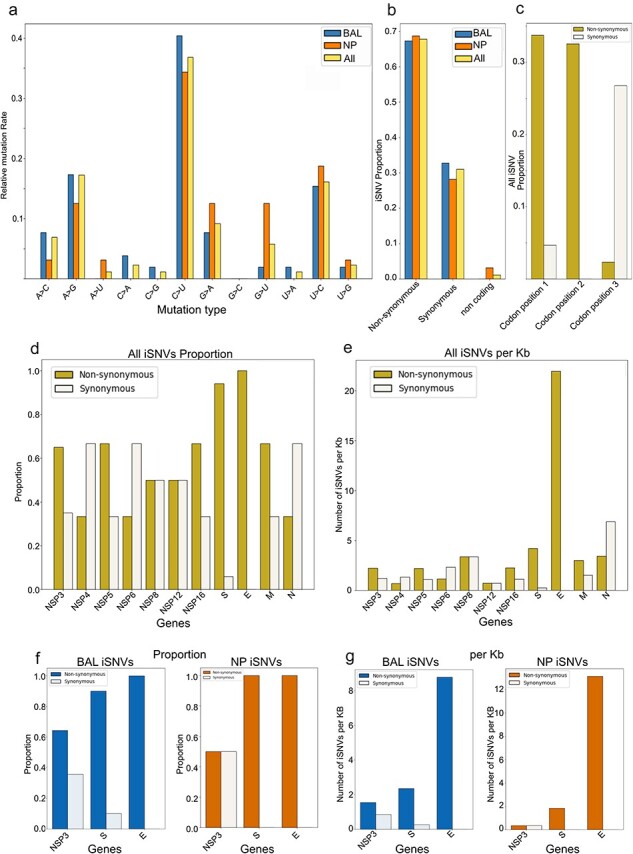
Intrahost variant impacts. (a) Mutation spectra for all iSNVs observed; (b) frequency of synonymous versus nonsynonymous mutations in BAL iSNVs, NP iSNVs, and all iSNVs combined; (c) synonymous and nonsynonymous amino acid changes at each codon position for all iSNVs observed; (d) frequency of synonymous versus nonsynonymous mutations, specifically in viral proteins with three or more iSNVs; (e) Quantification of iSNV per kilobase in viral proteins with three or more iSNV; (f) frequency of synonymous versus nonsynonymous mutations in BAL and NP for the three viral proteins (NPS3, spike, and envelope); (g) synonymous versus nonsynonymous mutations in BAL and NP for the three viral proteins (NPS3, spike, and envelope), represented as iSNVs per kilobase.

The iSNV mutational landscape was dominated by C–U transitions (Fig. 4a), which is in line with previous reports (Tonkin-Hill et al. 2021, Yi et al. 2021, Chaguza et al. 2023). Despite treatment with molnupiravir (Sanderson et al. 2023) the frequency of G-to-A iSNV was similar to that of the G-to-A SNVs seen in ancestral BF.7 (Supplementary Fig. S5). When we compared the mutation patterns between samples collected at different anatomic sites, we did not observe a marked difference between BAL and NP samples (Fig. 4a). Additionally, the frequency of synonymous and nonsynonymous mutations in iSNVs was similar in BAL and NP samples (Fig. 4b). As expected, most amino acid substitutions were concentrated in the first and second codons (Fig. 4c). Considering all iSNVs, we further examined the proportion and number per kilobase of synonymous versus nonsynonymous changes in SARS-CoV-2 proteins impacted by at least three iSNVs. The spike and envelope showed the highest frequency of nonsynonymous changes (Fig. 4d–e). However, only the envelope showed statistically significant enrichment for nonsynonymous changes (*P* = .000802) when the numbers were adjusted for gene length. We then investigated NP- and BAL-specific iSNVs for the three SARS-CoV-2 proteins with the highest number of variants. We observed a similar pattern for spike and envelope in both BAL and NP samples. For NSP3 in the NP samples, there was no difference in the frequency or the number of synonymous and nonsynonymous changes, while in the BAL sample, there was a modest difference (Fig. 4f–g). Deep mutational scanning of the complete XBB.1.5 spike reported by Dadonaite et al. (2024) suggests that key changes in the BAL spike include F375S and E619A, both of which are predicted to increase ACE2 binding (Dadonaite et al. 2024). Conversely, the substitution T523I in NP samples was predicted to enhance ACE2 binding, while the substitution V976A may contribute to immune escape, although at the expense of ACE2 binding (Dadonaite et al. 2024) (Supplementary Table S3).

### ORF8 knockouts were also observed in acute infections

The frameshift identified in the BAL sample and the large deletion detected in the NP sample resulted in loss of function (LOF) mutations within ORF8. During the routine genomic surveillance of SARS-CoV-2, we have noticed large putative deletions in the ORF7a/b/8 region (discerned through a lack of coverage in the region and, in some instances, via reads spanning the breakpoint). To confirm that such deletions can be found in acute infections, we performed long-range PCR on two routine samples suspected to have these deletions. The sequencing revealed an XBB.1.5 sublineage carrying a 306-bp deletion within ORF8 and a CH.1.1 sublineage carrying an 872-bp deletion, which removes ORF7a, ORF7b, and ORF8 (Supplementary Fig. S6). We used UShER to place these genomes on the SARS-CoV-2 phylogenetic tree and found that most of the adjacent genomes had either a gap or Ns in the same region (Supplementary Fig. S7), strongly indicating the presence of the respective deletions.

## Discussion

The case reported above documents a persistent SARS-CoV-2 infection in a patient undergoing B-cell depletion therapy with anti-CD20 therapy for DLBCL. The chronic infection was confirmed through migratory lung infiltrates and positive RT-PCR tests from samples collected in the upper and lower respiratory tracts. Determining the exact duration of chronic infection in this patient is a challenge, as sequencing of samples only began in June 2023, and the patient intermittently tested negative for SARS-CoV-2. Nonetheless, we could confirm via sequencing that the infection endured for >3 months (between June and September 2023), and based on the archaic lineage observed in the viral sequences, it is likely that the infection was acquired in June 2022. These findings suggest a prolonged infection lasting over a year and highlight the difficulty in diagnosing a low-grade SARS-CoV-2 viral infection in an immunocompromised patient. One of the strengths of this study is the examination of multiple samples from different anatomic compartments and sampling at various time points, providing a broader understanding of the infection. A chronic infection could potentially be missed if data were only based on samples collected from the same site and over shorter periods. The extended reconstitution period of the B-cell population after rituximab treatment (6–12 months), leading to significantly impaired autologous B-cell function, stands out as one of the most influential risk factors for prolonged SARS-CoV-2 infection and viral intrahost evolution among immunocompromised individuals (Van der Moeren et al. 2022, Raglow et al. 2024). Consequently, this population represents a crucial target for genetic surveillance of viral evolution in immunocompromised patients.

In this study, we obtained sequence data from eight positive specimens, covering six time points, including six NP samples, one ETA sample, and one BAL sample. The mutational pattern showed a predominance of C–U transitions, which is in line with the pattern of substitutions commonly observed in SARS-CoV-2 (Popa et al. 2020, Tonkin-Hill et al. 2021). While the patient received molnupiravir before sample collection, there was no characteristic excess of G-to-A mutations observed in the iSNV (Sanderson et al. 2023), suggesting that this treatment did not have a major impact on the evolution of the viral population in the patient. Furthermore, we observed a higher proportion of nonsynonymous changes relative to synonymous changes in the spike and envelope (although this difference only reached statistical significance for the envelope when adjusted for gene length), with no discernible difference between NP and BAL specimens.

The interpretation of amino acid changes is commonly based on their deviation from the Wuhan-Hu-1 reference sequence. However, following ∼4 years of selection and drift, the lineages that now dominate differ from the reference genome at a number of positions across the viral genome, especially in regions coding for viral proteins that are targeted by the host adaptive immune system. Like Omicron lineages BA.2 and BA.5, the BF.7 sublineage carries the amino acid change S375F in the spike RBD. While the S375F mutation was still present in NP samples, the sequence obtained from the BAL sample reverted to the wild-type sequence. Recently, deep mutational scanning using the XBB.1.5 and BA.2 spikes by Dadonaite et al. (2024) showed that a number of amino acid changes at Position 375 (including the reversion to the reference) can increase ACE2 binding and highlights that the impact of an amino acid change is often context dependent. Among the remaining 15 amino acid changes observed in spike, E619A (BAL) and T523I (NP) are predicted to increase ACE2 binding, while V976A (NP) facilitates immune escape but reduces ACE2 binding (Supplementary Table S3). S:P330S (BAL) is predicted to cause a modest increase in ACE2 binding and has been observed in other chronic infections involving immunosuppressed individuals (Kemp et al. 2021, Wilkinson et al. 2022, Dadonaite et al. 2024). Most of the spike amino acid changes are currently observed at a low frequency in Global Initiative on Sharing All Influenza Data (GISAID) (Supplementary Table S3). However, if we use persistent infections involving pre-VOC lineages as a guide for Omicron persistent infections, it is reasonable to assume that some of the amino acid changes observed in the current case may appear in future lineages that rise to prominence, especially if these lineages are derived from a BA.5/4 ancestor.

The spike protein plays a central role in cellular entry and represents a primary target for the adaptive immune system. Consequently, this protein has been subject to extensive scrutiny relative to the other 14 open reading frames of SARS-CoV-2. However, there is clear evidence that genetic variation outside the spike protein plays a role in the pathogenicity and fitness of SARS-CoV-2. Recent work using a recombinant SARS-CoV-2 consisting of the spike of Omicron (BA.1) and the backbone of an ancestral isolate showed higher virulence than BA.1, with mutations in nsp6 identified as the main driver of this difference in phenotype (Chen et al. 2023). It has also been noted that BA.5 outcompeted BA.4 despite having identical spike proteins (Parsons and Acharya 2023). Among the amino acid changes observed outside the spike in the current case, several have previously been associated with chronic infections. Notably, E:T30I has been observed in multiple chronic infections (Harari et al. 2024) but is rarely seen in circulating isolates (0.01% of GISAID genomes in January 2024) and appears to have a negative impact on the viruses’ ability to transmit to new hosts (Bloom and Neher 2023, Harari et al. 2024). Likewise, H125Y in the M protein has also been identified in multiple chronic infections (Wilkinson et al. 2022) and has been observed to arise in cell culture settings (Sonnleitner et al. 2022). It is infrequent in sequenced genomes (0.1% in GISAID as of January 2024), yet computational analyses suggest a fitness advantage for viruses harbouring this change (Wilkinson et al. 2022, Bloom and Neher 2023). Unlike the aforementioned amino acid changes, NSP3:K977Q has previously been observed in a VOC (Gamma, P.1) and has been detected in the context of chronic infections as well as within cryptic lineages sampled from wastewater (Faria et al. 2021, Gregory et al. 2022, Nabieva et al. 2023). This amino acid change enhances the activity of the SARS-CoV-2 papain-like protease, potentially modulating the host’s innate immune response (Patchett et al. 2021). Finally, while NSP5:K90R has not been implicated in chronic infections, of the amino acid changes observed in the patient, it was the one with the highest impact on fitness based on the work of Bloom and Neher (2023) (Supplementary Table S2), and like NSP3:K977Q, it may play a role in modulating the innate immune response (Fung et al. 2021). These findings underscore the diverse impacts of nonspike protein changes on viral transmission dynamics and host immune system modulation.

Beyond single amino acid changes, sequencing of NP and BAL samples revealed a large deletion and a frameshift mutation in the ORF8 region, resulting in a complete LOF of the viral accessory protein. Notably, ORF8 is recognized as one of the most hypervariable regions in the SARS-CoV-2 genome and presents the highest mutation density among nonstructural proteins (Bloom and Neher 2023). Since the early stages of the COVID-19 pandemic, various large deletions affecting ORF8 have been documented, with some found to reduce COVID-19 severity (Su et al. 2020, Young et al. 2020, Mazur-Panasiuk et al. 2021, Zinzula 2021, Tang et al. 2023). The mutational landscape of ORF8 over the past ∼4 years of the pandemic suggests a strong selective pressure driving rapid evolution, leading to ORF8 knockouts across multiple SARS-CoV-2 lineages (Gong et al. 2020, Su et al. 2020, Young et al. 2020, Mazur-Panasiuk et al. 2021). Several studies have reported evidence of relaxed purifying selection acting on ORF8, along with other accessory proteins (Bloom and Neher 2023), with ORF8 knockout clusters exhibiting a growth rate advantage (Wagner et al. 2024). Recent research by Kim and colleagues have proposed a biological mechanism underlying positive selection for ORF8 knockouts (Kim et al. 2023). They demonstrated that ORF8 can downregulate virion spike protein levels, thereby facilitating immune evasion by reducing the presence of spike on the surface of infected cells (Kim et al. 2023). Conversely, the absence of ORF8 increases spike protein levels on the virion, potentially enhancing its ability to infect host cells. This dynamic may confer a net benefit depending on the context. In immunocompromised individuals, viral lineages lacking ORF8 could have ‘the best of both worlds’, increasing virion infectivity without paying the price of increased immune recognition of infected cells. Moreover, viral lineages lacking a complete ORF8 have been reported in immunocompromised patients with chronic SARS-CoV-2 infections (Van Cleemput et al. 2021, Guilbaud et al. 2024), underscoring the importance of detecting ORF8 knockouts in SARS-CoV-2 genomic surveillance efforts.

Our phylogenetic analysis revealed that NP samples formed a cohesive cluster, while BAL and ETA samples occupied a branch that diverged from NP samples early on, indicating a prolonged period of independent evolution. Notably, in the initial samples collected in July 2023, the BAL sample carried 23 iSNVs (allele frequency >87%) not seen in the NP sample. The NP sample carried 27 patient-specific variants (allele frequency >95%) that were not observed in the BAL sample. For reference, the original VOC Alpha had only 23 nucleotide changes compared to its ancestral lineage (Hill et al. 2022). Although significant divergence was observed between variants detected in NP and BAL samples, most of the iSNVs found in the ETA sample were also identified in NP or BAL samples. Intrahost divergence has been observed in acute SARS-CoV-2 infection through concurrent testing of saliva and NP swab samples (Farjo et al. 2024). In cases of chronic infection, more dramatic divergence has been observed. Van Cleemput et al. (2021) observed organ-specific SARS-CoV-2 lineages in postmortem samples obtained from patients who had died from COVID-19. In a comprehensive study, Chaguza et al. (2023) followed an immunocompromised patient suffering from B-cell lymphoma with a chronic infection involving the pre-VOC lineage B.1.517, which lasted in excess of 1 year. Using sequences generated from 30 longitudinal samplings, they were able to identify at least three distinct viral genotypes in the patient, suggesting that distinct viral populations in other anatomic regions were present in the patient, and they continually restocked the nasopharynx. In the current case, we see a similar phenomenon with the appearance of a distinct lineage in the nasopharynx, which is likely to have evolved in a different anatomic location. In contrast to Chaguza et al., we have a more limited number of NP samples covering a much shorter time period and observed only a single lineage in the nasopharynx. However, we did see the appearance and disappearance of iSNV with allele frequencies of over 80% between samplings, which may suggest a seeding of the nasopharynx from a distinct location.

The results presented above contribute to growing evidence indicating that SARS-CoV-2 can be identified in various anatomic compartments (Stein et al. 2022, Bussani et al. 2023, Proal et al. 2023, Machkovech et al. 2024) and that these locations can serve as a viral reservoir, often going undetectable by conventional SARS-CoV-2 testing (Bussani et al. 2023). This highlights that further investigation on how these tissue reservoirs contribute to SARS-CoV-2 persistence is needed. These observations also have implications for the care of immunocompromised individuals, as a negative SARS-CoV-2 test is not a guarantee that an infection has cleared. Alternative specimen types, such as stool, should be considered (Machkovech et al. 2024), and any SARS-CoV-2-positive samples from immunocompromised patients should be sequenced to distinguish between acute and long-term SARS-CoV-2 infections.

We should close by mentioning that this work is subject to various inherent limitations, and notable caveats should be highlighted. Firstly, and most importantly, we do not have sequences from the patient’s positive tests in 2022, especially during the patient’s first hospitalization. As a consequence, we cannot definitively prove a chronic infection beyond the 85 days covered by the six NP samples. Additionally, we only had single samples for the BAL and ETA, which made it impossible to determine if the pattern of iSNV was typical of these anatomic compartments. Also, this makes it more difficult to rule out potential contamination, particularly for the ETA sample, as it contains a mix of iSNV found in the BAL and NP samples. Finally, it should be mentioned that while we see divergent lineages in the different samples, there is no indication that these are the anatomic compartments in which these lineages evolved. Based on the negative PCR results from late 2022 to early 2023, including those from a BAL sample, we can conclude that SARS-CoV-2 was absent from the NP and at least part of the lung during this period. Nevertheless, we believe that the scenario of a chronic infection lasting over a year is the most parsimonious explanation for the available data.

## Materials and methods

### SARS-CoV-2 genomic surveillance at the CHU of Liège

Since the beginning of the pandemic, the Department of Human Genetics, in collaboration with the Department of Clinical Microbiology of the CHU of Liège (Belgium), has been following the evolution of SARS-CoV-2 lineages circulating the region (Artesi et al. 2020, Bollen et al. 2021, Dellicour et al. 2021). We have been a member of the National Genomic Surveillance Platform for SARS-CoV-2 in Belgium since its inception in 2021 (Cuypers et al. 2022). The samples used in the current study were initially collected and sequenced as a part of the Belgian SARS-CoV-2 genomic surveillance effort.

### Real-time PCR and RNA extraction

In most cases, SARS-CoV-2 was detected in patient samples via the Cobas 6800 platform (Roche) using the Cobas SARS-CoV-2 real-time PCR assay, which detects the ORF1ab and E genes. Alternatively, the Alinity SARS-CoV-2 assay (Abbott) or Genexpert SARS-CoV-2 assay (Cepheid) was used. Positive RNA was extracted from the NP swab samples or BAL (300 µl) with the Maxwell 48 device and the Maxwell RSC Viral TNA kit (Promega) following the manufacturer’s instructions.

### SARS-CoV-2 whole-genome sequencing

The samples sequenced had a *C*_t_ that ranged between 19 and 26.4 (median 20). For RT, we utilized 3.3 µl of eluted RNA, 1.2 µl of SuperScript IV VILO™ Master Mix, and 1.5 µl of H_2_O for a total volume of 6 µl. The mix was incubated at 25°C for 10 min, 50°C for 10 min, and 85°C for 5 min. PCR was carried out using Q5® High-Fidelity DNA Polymerase (New England Biolabs) and the ARTIC v5.2.0 1200-bp amplicon primer pools (https://github.com/quick-lab/SARS-CoV-2/tree/main/1200/v5.2.0_1200). PCR conditions were set up according to the recommendations of the ARTIC network sequencing protocol (https://www.protocols.io/view/ncov-2019-sequencing-protocol-v3-locost-bp2l6n26rgqe/v3).

The samples were sequenced twice. The first sequencing was conducted as part of routine SARS-CoV-2 surveillance. In this case, the PCR products were indexed using the Nanopore Rapid Barcoding Kit 96 V14 (SQK-RBK114.96) in the same manner described in Freed et al. (2020). Sequencing was carried out on a GridION using 10.4.1 flow cells, with Super-accurate basecalling, carried out using Guppy 6.5.7. The replicate sequencing also used the ARTIC v5.2.0 1200-bp amplicon primer pools, and indexing was done using the Native Barcoding Kit 96 V14 (SQK-NBD114.96) following the manufacturer’s instructions. Sequencing used 10.4.1 flow cells on the GridION, and demultiplexing required barcodes on both ends of the molecule. Basecalling was carried out with dorado-0.5.2 (https://github.com/nanoporetech/dorado) using the dna_r10.4.1_e8.2_400bps_sup@v4.3.0 model. We also used the duplex pipeline with the dna_r10.4.1_e8.2_400bps_sup@v4.3.0 model; duplex reads were extracted from the resultant Binary Alignment Map (BAM) file using SAMtools. Sequencing adapters were removed using Porechop (https://github.com/rrwick/Porechop), and subsequently, primers were removed by trimming 40 bases from both ends of the read with NanoFilt (https://github.com/wdecoster/nanofilt).

### Long-range PCR and sequencing

In the samples carrying a deletion in the ORF7/8 region, we carried out long-range PCR with the primers 27_LEFT (5ʹ-TGGATCACCGGTGGAATTGCTA-3ʹ) and 28_RIGHT (5ʹ-GTTTGGCCTTGTTGTTGTTGGC-3ʹ) from the study by Freed et al. (2020). RT and PCR conditions were the same as those used for the ARTIC whole-genome amplicon sequencing protocol. For these PCR products to retain the full-length amplicon, we used the Native Barcoding Kit 96 V14 (SQK-NBD114.96), which ligates barcodes to the ends of the amplicon. Sequencing was also carried out on a GridION using 10.4.1 flow cells, with Super-accurate basecalling carried out using Guppy 6.5.7. The replicate long-range PCR was base-called in the same manner as the ARTIC v5.2.0 1200-bp amplicon pools.

### Variant calling and consensus genome generation

Consensus genomes were generated using the ARTIC network field bioinformatics pipeline (https://github.com/artic-network/fieldbioinformatics) with high-quality duplex reads. In the consensus sequences, iSNVs with an allele frequency of >80% are incorporated into the consensus. If the allele frequency is between 50% and 80%, the nucleotide position is represented as N. For iSNVs with an allele frequency <50%, the reference nucleotide is retained. Additionally, SNVs were called using LoFreq (Wilm et al. 2012), while indels were called using the ARTIC network pipeline as well as Clair3 (Zheng et al. 2022). The allele frequencies reported were generated by LoFreq using the high-quality duplex reads. The SNVs with an allele frequency of >98% in all samples were classified as ancestral BF.7 SNVs. The one exception was the SNV C27889T that was encompassed by the 362-bp deletion in the NP and ETA samples, but as it is found in 95% of BF.7 samples, it was classified as an ancestral SNV. iSNVs were required to have an allele frequency of >19% in at least one sample and be present in both replicates for that sample, as well as in the simplex and duplex reads. To be included, an iSNV needed at least 100 supporting reads in the resequencing. Total coverage at the position where each iSNV was called is given in Supplementary Table S4. Variant Call Format files (VCFs) and BAMs were checked in the Integrative Genomics Viewer (Thorvaldsdóttir et al. 2013). The consensus sequence for the long-range PCR products was generated using the bcftools consensus command (https://samtools.github.io/bcftools/bcftools.html) and manually incorporated into the ARTIC network field bioinformatics pipeline consensus.

In order to generate the patient BF.7 ancestral sequence, we used the bcftools isec tool to select SNVs and deletions with an allele frequency of >98% that are common to all patient samples. We also included an SNV found in the BAL and ETA samples, which overlaps with the 362-bp deletion in the NP samples. The resulting VCF file contains 72 SNVs/deletions, and this file was used in combination with the bcftools consensus tool to produce a consensus sequence for the ancestral lineage.

### Phylogenetics

Consensus genomes were placed on the existing phylogenetic tree via UShER using the phylogenetic tree generated with genomes from GISAID, GeneBank, COVID-19 Genomics UK Consortium, and China National Center for Bioinformation. In the case of the BF.7 samples, we used the genomes identified by UShER to infer a time-scaled phylogenetic tree using the software package BEAST v1.10.5 (Suchard et al. 2018) with the following settings: a skygrid demographic model, a relaxed molecular clock with a normal (8 × 10^−4^, 1 × 10^−4^) prior distribution, and an HKY + G4 + I substitution model (Hasegawa et al. 1985, Yang 1994). Three independent Markov chain Monte Carlo runs were performed, each running for 10^6^ iterations and sampling every 10^4^ states. Convergence and mixing of all parameters were assessed using Tracer v1.7 (Rambaut et al. 2018), and a maximum clade credibility tree was obtained using TreeAnnotator v.1.10.5 (Suchard et al. 2018) after combining all results using LogCombiner v.1.10.5 (Suchard et al. 2018) and discarding the first 10% of samples as burn-in.

### Statistical analysis

Statistical analysis was carried out in Python (version 3.9.16) using pandas (version 1.4.2) and stats models (version 0.14.0). We tested for a difference in the frequency of synonymous versus nonsynonymous changes in genes with three or more unique iSNVs, adjusted by gene length, with Fisher’s exact test; *P*-values were adjusted for multiple testing using the Benjamini–Hochberg procedure.

### Ethics statement

Methods of collection, testing of biological specimens, and protected health information used in this study were approved by the University Hospital of Liège Ethics Review Board, reference number: 2020-139; informed consent was obtained from the patient.

## Supplementary Material

veae073_Supp

## Data Availability

The consensus SARS-CoV-2 genomes and raw reads are available on GISAID (www.gisaid.org) under accession numbers EPI_ISL_17960749, EPI_ISL_18515316, EPI_ISL_17960747, EPI_ISL_18059076, EPI_ISL_18059074, EPI_ISL_18059075, EPI_ISL_18136392, and EPI_ISL_18274346. All GISAID genomes used in the analysis can be found via EPI_SET ID: EPI_SET_240229zf, doi:10.55876/gis8.240229zf. BAM files for each sample, including both sequencing runs, have also been deposited on the European Nucleotide Archive (ENA) under the accession numbers ERS18346681, ERS18346682, ERS18346683, ERS18346684, ERS18346685, ERS18346686, ERS18346687, and ERS18346688. The file names in ENA reflect the GISAID IDs.

## References

[R1] Artesi M , Bontems S, Göbbels P et al. A recurrent mutation at position 26340 of SARS-CoV-2 is associated with failure of the E gene quantitative reverse transcription-PCR utilized in a commercial dual-target diagnostic assay. *J Clin Microbiol* 2020;58:10–128. doi: 10.1128/JCM.01598-20 PMC7512182 32690547

[R2] Avanzato VA , Matson MJ, Seifert SN et al. Case study: prolonged infectious SARS-CoV-2 shedding from an asymptomatic immunocompromised individual with cancer. *Cell* 2020;183:1901–12.e9. doi: 10.1016/j.cell.2020.10.049 33248470 PMC7640888

[R3] Bloom JD , Neher RA. Fitness effects of mutations to SARS-CoV-2 proteins. *Virus Evol* 2023;9:vead055. doi: 10.1093/ve/vead055 PMC10506532 37727875

[R4] Bollen N , Artesi M, Durkin K et al. Exploiting genomic surveillance to map the spatio-temporal dispersal of SARS-CoV-2 spike mutations in Belgium across 2020. *Sci Rep* 2021;11:18580. doi: 10.1038/s41598-021-97667-9 PMC8448849 34535691

[R5] Bussani R , Zentilin L, Correa R et al. Persistent SARS-CoV-2 infection in patients seemingly recovered from COVID-19. *J Pathol* 2023;259:254–63. doi: 10.1002/path.6035 36651103 PMC10107739

[R6] Carabelli AM , Peacock TP, Thorne LG et al. SARS-CoV-2 variant biology: immune escape, transmission and fitness. *Nat Rev Microbiol* 2023;21:162–77. doi: 10.1038/s41579-022-00841-7 36653446 PMC9847462

[R7] Chaguza C , Hahn AM, Petrone ME et al. Accelerated SARS-CoV-2 intrahost evolution leading to distinct genotypes during chronic infection. *Cell Rep Med* 2023;4:100943. doi: 10.1016/j.xcrm.2023.100943 PMC9906997 36791724

[R8] Chen D-Y , Chin CV, Kenney D et al. Spike and nsp6 are key determinants of SARS-CoV-2 Omicron BA.1 attenuation. *Nature* 2023;615:143–50. doi: 10.1038/s41586-023-05697-2 36630998

[R9] Choi B , Choudhary MC, Regan J et al. Persistence and evolution of SARS-CoV-2 in an immunocompromised host. *New Engl J Med* 2020;383:2291–93. doi: 10.1056/NEJMc2031364 33176080 PMC7673303

[R10] Cuypers L , Dellicour S, Hong SL et al. Two years of genomic surveillance in Belgium during the SARS-CoV-2 pandemic to attain country-wide coverage and monitor the introduction and spread of emerging variants. *Viruses* 2022;14:2301. doi: 10.3390/v14102301 PMC9612291 36298856

[R11] Dadonaite B , Brown J, McMahon TE et al. Spike deep mutational scanning helps predict success of SARS-CoV-2 clades. *Nature* 2024;631:617–26. doi: 10.1038/s41586-024-07636-1 38961298 PMC11254757

[R12] Dellicour S , Durkin K, Hong SL et al. A phylodynamic workflow to rapidly gain insights into the dispersal history and dynamics of SARS-CoV-2 lineages. *Mol Biol Evol* 2021;38:1608–13. doi: 10.1093/molbev/msaa284 33316043 PMC7665608

[R13] Faria NR , Mellan TA, Whittaker C et al. Genomics and epidemiology of the P.1 SARS-CoV-2 lineage in Manaus, Brazil. *Science* 2021;372:815–21. doi: 10.1126/science.abh2644 33853970 PMC8139423

[R14] Farjo M , Koelle K, Martin MA et al. Within-host evolutionary dynamics and tissue compartmentalization during acute SARS-CoV-2 infection. *J Virol* 2024;98:e0161823. doi: 10.1128/jvi.01618-23 PMC10805032 38174928

[R15] Feng A , Bevins S, Chandler J et al. Transmission of SARS-CoV-2 in free-ranging white-tailed deer in the United States. *Nat Commun* 2023;14:4078. doi: 10.1038/s41467-023-39782-x PMC10333304 37429851

[R16] Freed NE , Vlková M, Faisal MB et al. Rapid and inexpensive whole-genome sequencing of SARS-CoV-2 using 1200 bp tiled amplicons and Oxford Nanopore Rapid Barcoding. *Biol Methods Protoc* 2020;5:bpaa014. doi: 10.1093/biomethods/bpaa014 PMC7454405 33029559

[R17] Fung S-Y , Siu K-L, Lin H et al. SARS-CoV-2 main protease suppresses type I interferon production by preventing nuclear translocation of phosphorylated IRF3. *Int J Bio Sci* 2021;17:1547–54. doi: 10.7150/ijbs.59943 33907518 PMC8071772

[R18] Gong Y-N , Tsao K-C, Hsiao M-J et al. SARS-CoV-2 genomic surveillance in Taiwan revealed novel ORF8-deletion mutant and clade possibly associated with infections in Middle East. *Emerging Microbes Infect* 2020;9:1457–66. doi: 10.1080/22221751.2020.1782271 PMC7473175 32543353

[R19] Gregory DA , Trujillo M, Rushford C et al. Genetic diversity and evolutionary convergence of cryptic SARS- CoV-2 lineages detected via wastewater sequencing. *PLoS Pathogens* 2022;18:e1010636. doi: 10.1371/journal.ppat.1010636 PMC9604950 36240259

[R20] Guilbaud R , Franco Yusti A-M, Leducq V et al. Higher levels of SARS-CoV-2 genetic variation in immunocompromised patients: a retrospective case-control study. *J Infect Dis* 2024;229:1041–49. doi: 10.1093/infdis/jiad499 37956413

[R21] Harari S , Miller D, Fleishon S et al. Using big sequencing data to identify chronic SARS-Coronavirus-2 infections. *Nat Commun* 2024;15:648. doi: 10.1038/s41467-024-44803-4 PMC10799923 38245511

[R22] Hasegawa M , Kishino H, Yano T. Dating of the human-ape splitting by a molecular clock of mitochondrial DNA. *J Mol Evol* 1985;22:160–74. doi: 10.1007/bf02101694 3934395

[R23] Hill V , Du Plessis L, Peacock TP et al. The origins and molecular evolution of SARS-CoV-2 lineage B.1.1.7 in the UK. *Virus Evol* 2022;8:veac080. doi: 10.1093/ve/veac080 PMC9752794 36533153

[R24] Hui KPY , Ho JCW, Cheung M-C et al. SARS-CoV-2 Omicron variant replication in human bronchus and lung ex vivo. *Nature* 2022;603:715–20. doi: 10.1038/s41586-022-04479-6 35104836

[R25] Jung C , Kmiec D, Koepke L et al. Omicron: what makes the latest SARS-CoV-2 variant of concern so concerning? *J Virol* 2022;96:e0207721. doi: 10.1128/jvi.02077-21 PMC8941872 35225672

[R26] Kaku Y , Okumura K, Padilla-Blanco M et al. Virological characteristics of the SARS-CoV-2 JN.1 variant. *Lancet Infect Dis* 2024;24:e82. doi: 10.1016/S1473-3099(23)00813-7 38184005

[R27] Kemp SA , Collier DA, Datir RP et al. SARS-CoV-2 evolution during treatment of chronic infection. *Nature* 2021;592:277–82. doi: 10.1038/s41586-021-03291-y 33545711 PMC7610568

[R28] Kim I-J , Lee Y-H, Khalid MM et al. SARS-CoV-2 protein ORF8 limits expression levels of Spike antigen and facilitates immune evasion of infected host cells. *J Biol Chem* 2023;299:104955. doi: 10.1016/j.jbc.2023.104955 PMC10289268 37354973

[R29] Machkovech HM , Hahn AM, Garonzik Wang J et al. Persistent SARS-CoV-2 infection: significance and implications. *Lancet Infect Dis* 2024;24:e453–62. doi: 10.1016/S1473-3099(23)00815-0 38340735

[R30] Markov PV , Ghafari M, Beer M et al. The evolution of SARS-CoV-2. *Nat Rev Microbiol* 2023;21:361–79. doi: 10.1038/s41579-023-00878-2 37020110

[R31] Mazur-Panasiuk N , Rabalski L, Gromowski T et al. Expansion of a SARS-CoV-2 Delta variant with an 872 nt deletion encompassing ORF7a, ORF7b and ORF8, Poland, July to August 2021. *Euro Surveillance* 2021;26. doi: 10.2807/1560-7917.ES.2021.26.39.2100902 PMC8485581 34596017

[R32] Nabieva E , Komissarov AB, Klink GV et al. A highly divergent SARS-CoV-2 lineage B.1.1 sample in a patient with long-term COVID-19. *MedRxiv* 2023. doi: 10.1101/2023.09.14.23295379 PMC12491319 41050757

[R33] Obermeyer F , Jankowiak M, Barkas N et al. Analysis of 6.4 million SARS-CoV-2 genomes identifies mutations associated with fitness. *Science* 2022;376:1327–32. doi: 10.1126/science.abm1208 35608456 PMC9161372

[R34] Parsons RJ , Acharya P. Evolution of the SARS-CoV-2 Omicron spike. *Cell Rep* 2023;42:113444. doi: 10.1016/j.celrep.2023.113444 PMC10782855 37979169

[R35] Patchett S , Lv Z, Rut W et al. A molecular sensor determines the ubiquitin substrate specificity of SARS-CoV-2 papain-like protease. *Cell Rep* 2021;36:109754. doi: 10.1016/j.celrep.2021.109754 PMC8423903 34547223

[R36] Pather S , Madhi SA, Cowling BJ et al. SARS-CoV-2 Omicron variants: burden of disease, impact on vaccine effectiveness and need for variant-adapted vaccines. *Front Immunol* 2023;14:1130539. doi: 10.3389/fimmu.2023.1130539 PMC10242031 37287979

[R37] Popa A , Genger J-W, Nicholson MD et al. Genomic epidemiology of superspreading events in Austria reveals mutational dynamics and transmission properties of SARS-CoV-2. *Sci Trans Med* 2020;12:eabe2555. doi: 10.1126/scitranslmed.abe2555 PMC7857414 33229462

[R38] Proal AD , VanElzakker MB, Aleman S et al. SARS-CoV-2 reservoir in post-acute sequelae of COVID-19 (PASC). *Nat Immunol* 2023;24:1616–27. doi: 10.1038/s41590-023-01601-2 37667052

[R39] Raglow Z , Surie D, Chappell JD et al. SARS-CoV-2 shedding and evolution in patients who were immunocompromised during the omicron period: a multicentre, prospective analysis. *Lancet Microbe* 2024;5:e235–46. doi: 10.1016/S2666-5247(23)00336-1 38286131 PMC11849777

[R40] Rambaut A , Drummond AJ, Xie D et al. Posterior summarization in Bayesian phylogenetics using tracer 1.7. *Syst Biol* 2018;67:901–04. doi: 10.1093/sysbio/syy032 29718447 PMC6101584

[R41] Roemer C , Sheward DJ, Hisner R et al. SARS-CoV-2 evolution in the Omicron era. *Nat Microbiol* 2023;8:1952–59. doi: 10.1038/s41564-023-01504-w 37845314

[R42] Sanderson T , Hisner R, Donovan-Banfield I et al. A molnupiravir-associated mutational signature in global SARS-CoV-2 genomes. *Nature* 2023;623:594–600. doi: 10.1038/s41586-023-06649-6 37748513 PMC10651478

[R43] Sonnleitner ST , Sonnleitner S, Hinterbichler E et al. The mutational dynamics of the SARS-CoV-2 virus in serial passages in vitro. *Virologica Sin* 2022;37:198–207. doi: 10.1016/j.virs.2022.01.029 PMC8800542 35277373

[R44] Stein SR , Ramelli SC, Grazioli A et al. SARS-CoV-2 infection and persistence in the human body and brain at autopsy. *Nature* 2022;612:758–63. doi: 10.1038/s41586-022-05542-y 36517603 PMC9749650

[R45] Su YCF , Anderson DE, Young BE et al. Discovery and genomic characterization of a 382-nucleotide deletion in ORF7b and ORF8 during the early evolution of SARS-CoV-2. *mBio* 2020;11:10–128. doi: 10.1128/mBio.01610-20 PMC7374062 32694143

[R46] Suchard MA , Lemey P, Baele G et al. Bayesian phylogenetic and phylodynamic data integration using BEAST 1.10. *Virus Evol* 2018;4:vey016. doi: 10.1093/ve/vey016 PMC6007674 29942656

[R47] Tang Z , Yu P, Guo Q et al. Clinical characteristics and host immunity responses of SARS-CoV-2 Omicron variant BA.2 with deletion of ORF7a, ORF7b and ORF8. *Virol J* 2023;20:106. doi: 10.1186/s12985-023-02066-3 PMC10226014 37248496

[R48] Thorvaldsdóttir H , Robinson JT, Mesirov JP. Integrative Genomics Viewer (IGV): high-performance genomics data visualization and exploration. *Briefings Bioinf* 2013;14:178–92. doi: 10.1093/bib/bbs017 PMC3603213 22517427

[R49] Tonkin-Hill G , Martincorena I, Amato R et al. Patterns of within-host genetic diversity in SARS-CoV-2. *eLife* 2021;10:e66857. doi: 10.7554/eLife.66857 PMC8363274 34387545

[R50] Turakhia Y , Thornlow B, Hinrichs AS et al. Ultrafast Sample placement on Existing tRees (UShER) enables real-time phylogenetics for the SARS-CoV-2 pandemic. *Nat Genet* 2021;53:809–16. doi: 10.1038/s41588-021-00862-7 33972780 PMC9248294

[R51] Van Cleemput J , van Snippenberg W, Lambrechts L et al. Organ-specific genome diversity of replication-competent SARS-CoV-2. *Nat Commun* 2021;12:6612. doi: 10.1038/s41467-021-26884-7 PMC8595628 34785663

[R52] Van der Moeren N , Selhorst P, Ha M et al. Viral evolution and immunology of SARS-CoV-2 in a persistent infection after treatment with rituximab. *Viruses* 2022;14:752. doi: 10.3390/v14040752 PMC9032773 35458482

[R53] Viana R , Moyo S, Amoako DG et al. Rapid epidemic expansion of the SARS-CoV-2 Omicron variant in southern Africa. *Nature* 2022;603:679–86. doi: 10.1038/s41586-022-04411-y 35042229 PMC8942855

[R54] Wagner C , Kistler KE, Perchetti GA et al. Positive selection underlies repeated knockout of ORF8 in SARS-CoV-2 evolution. *Nat Commun* 2024;15:3207. doi: 10.1038/s41467-024-47599-5 PMC11016114 38615031

[R55] Wilkinson SAJ , Richter A, Casey A et al. Recurrent SARS-CoV-2 mutations in immunodeficient patients. *Virus Evol* 2022;8:veac050. doi: 10.1093/ve/veac050 PMC9384748 35996593

[R56] Wilm A , Aw PPK, Bertrand D et al. LoFreq: a sequence-quality aware, ultra-sensitive variant caller for uncovering cell-population heterogeneity from high-throughput sequencing datasets. *Nucleic Acids Res* 2012;40:11189–201. doi: 10.1093/nar/gks918 23066108 PMC3526318

[R57] Yang Z . Maximum likelihood phylogenetic estimation from DNA sequences with variable rates over sites: approximate methods. *J Mol Evol* 1994;39:306–14. doi: 10.1007/bf00160154 7932792

[R58] Yi H , Wang J, Wang J et al. The emergence and spread of novel SARS-CoV-2 variants. *Front Public Health* 2021;9:696664. doi: 10.3389/fpubh.2021.696664 PMC8364952 34409009

[R59] Young BE , Fong S-W, Chan Y-H et al. Effects of a major deletion in the SARS-CoV-2 genome on the severity of infection and the inflammatory response: an observational cohort study. *Lancet* 2020;396:603–11. doi: 10.1016/S0140-6736(20)31757-8 32822564 PMC7434477

[R60] Zheng Z , Li S, Su J et al. Symphonizing pileup and full-alignment for deep learning-based long-read variant calling. *Nat Comput Sci* 2022;2:797–803. doi: 10.1038/s43588-022-00387-x 38177392

[R61] Zinzula L . Lost in deletion: the enigmatic ORF8 protein of SARS-CoV-2. *Biochem Biophys Res Commun* 2021;538:116–24. doi: 10.1016/j.bbrc.2020.10.045 33685621 PMC7577707

