# Supplementary material for: Intrahost evolution leading to distinct lineages in the upper and lower respiratory tracts during SARS-CoV-2 prolonged infection
Source: Virus Evol. 2024 Aug 31;10(1):veae073. doi: 10.1093/ve/veae073 (PMC11470753; doi:10.1093/ve/veae073)
Supplement: veae073_Supp [file veae073_supp.zip › suppl_data/Supplementary-Figures.pdf]

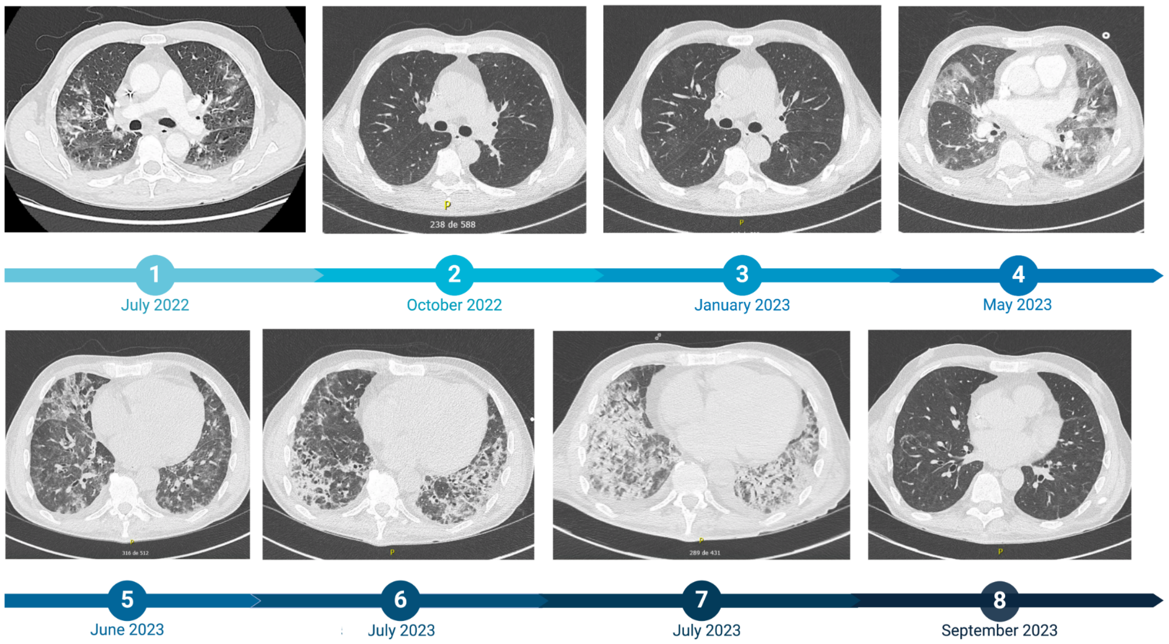

Supplementary Figure S1. Evolution of the lesions visualized with chest CT scan over a period of one year. 1, Contrast-enhanced axial chest CT images performed during the first hospitalization showed bilateral and diffuse ground-glass opacities, consistent with moderate COVID-19 pneumonia (involvement of 20 to 25% of lung volume), and bilateral pleural effusions; 2, Axial chest CT images performed three months later showed a decrease in the extent and density of bilateral ground-glass opacities, which persist in peripheral and subpleural areas, and resolution of pleural effusions, indicating a positive response to treatment; 3, Axial chest CT images showed a decrease in density and a slight reduction in the extent of bilateral ground-glass opacities; 4, Contrast-enhanced axial chest CT images performed during the second hospitalization showed extensive ground-glass opacities with significant densification in both lungs involving more than 75% of lung volume, and the appearance of a 14 mm thick right pleural effusion; 5, Axial chest CT images performed during the third hospitalization showed multiple bilateral ground-glass opacities, with areas showing hyperdense opacities and condensation with a retractile appearance, accompanied by traction bronchiectasis; 6, Axial chest CT images showed a slight intensification of bilateral ground-glass opacities, regression of consolidations in the upper lobes with increased bronchiectasis features in the lower lobes, alongside near-complete regression of pleural effusions, suggesting a discordant evolution of interstitial involvement with potential lower lobar infection; 7, Axial chest CT images showed a worsening of bilateral panlobar pneumonia with increased consolidation volume and size of confluence foci, alongside the emergence of a mild bilateral pleural effusion; 8, Axial chest CT images performed after the third hospitalization showed a decrease in bilateral ground-glass opacities and a reduction of consolidative opacities in the lower lobes, right upper lobe, and lingula, with less prominent bronchiectasis features.

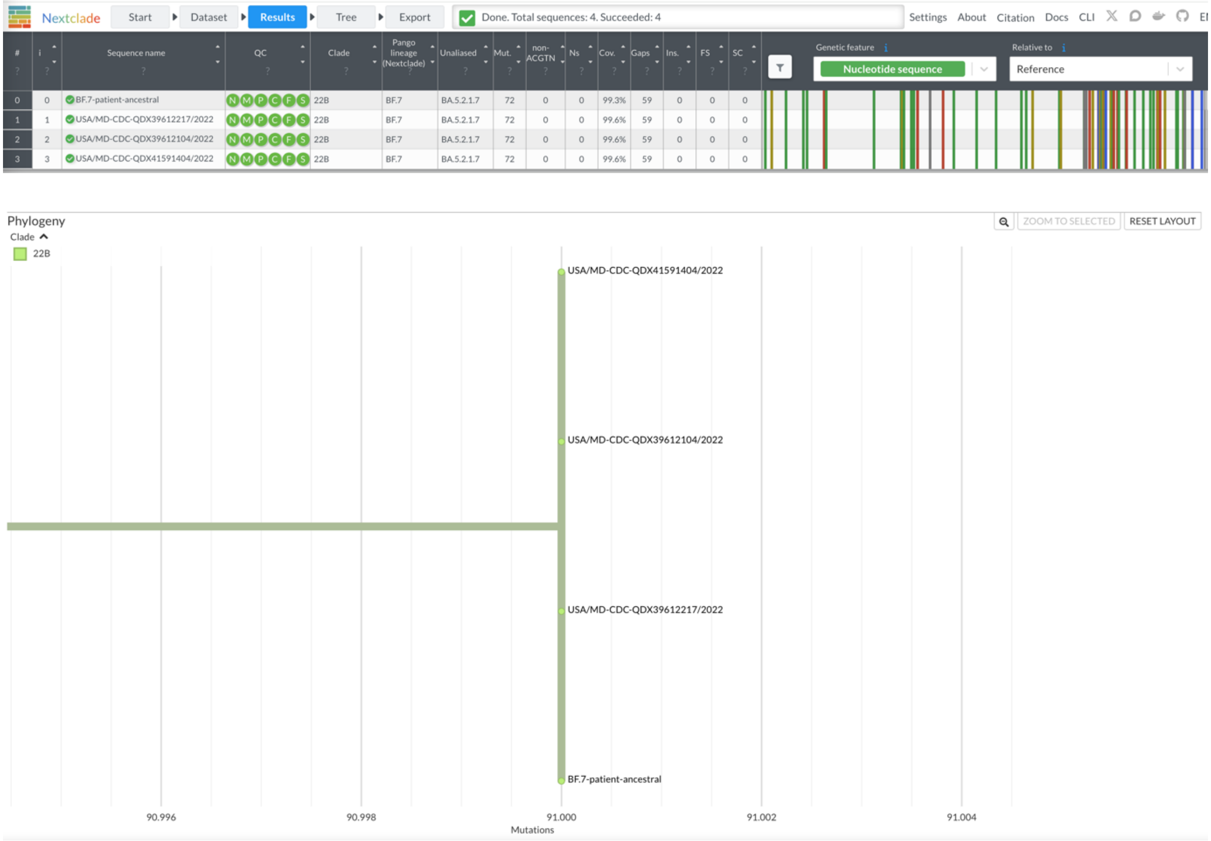

Supplementary Figure S2. Screen shots from Nextclade (https://clades.nextstrain.org/) shows the consensus genome reflecting the 72 SNVs/deletions fixed in the eight samples matched three BF.7 genomes.

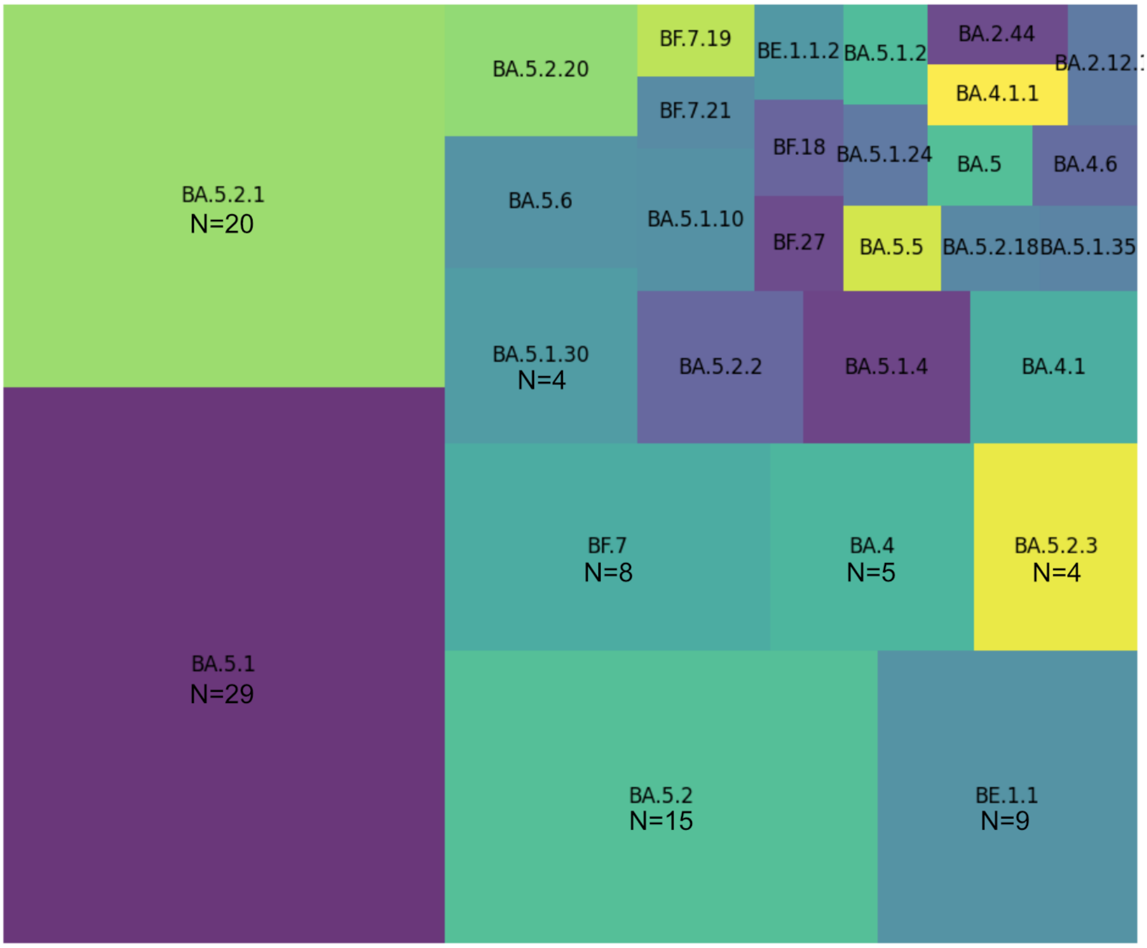

Supplementary Figure S3. Lineages observed in Liège, Belgium during the week of the patients 1st hospitalisation for COVID-19 pneumopathy, July 2022.

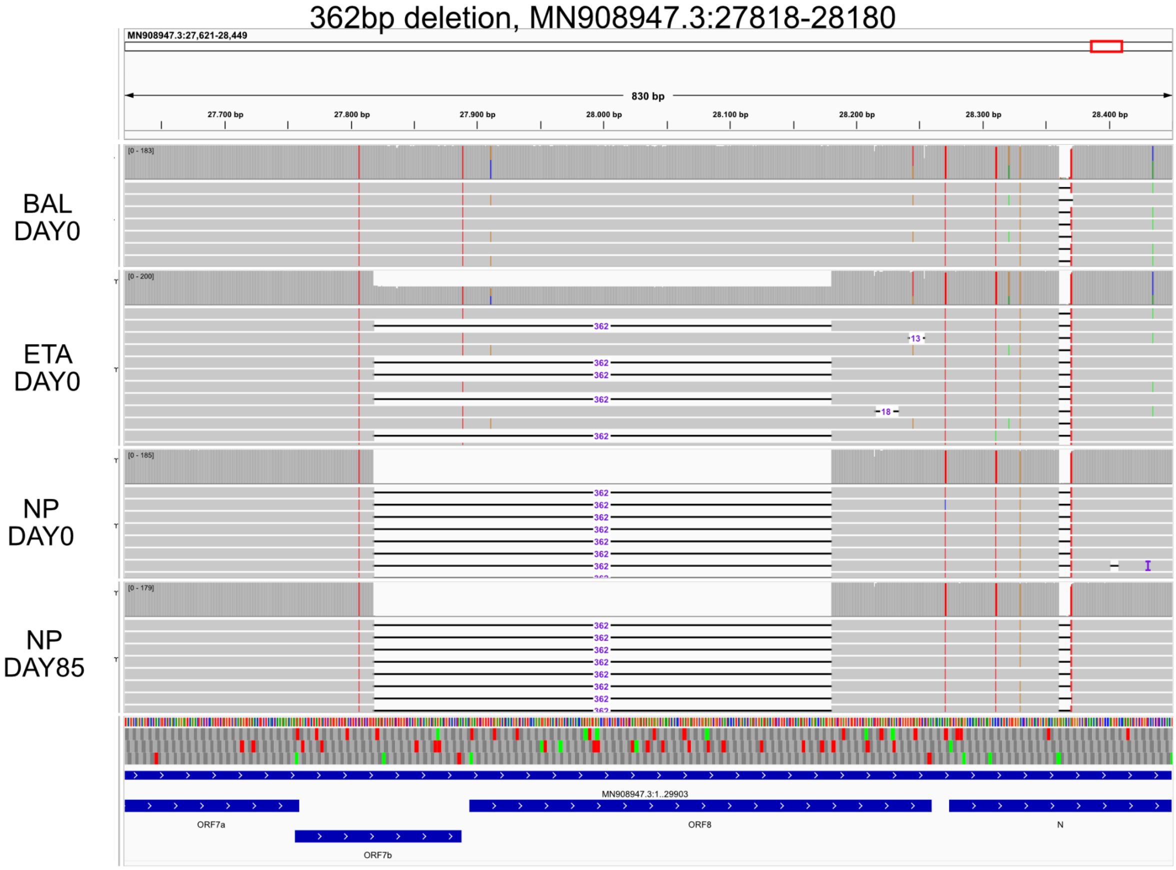

Supplementary Figure S4. Long range PCR and nanopore sequencing of the 362bp deletion impacting ORF7b and ORF8 observed in the NP sample and a subset of the reads in the ETA sample.

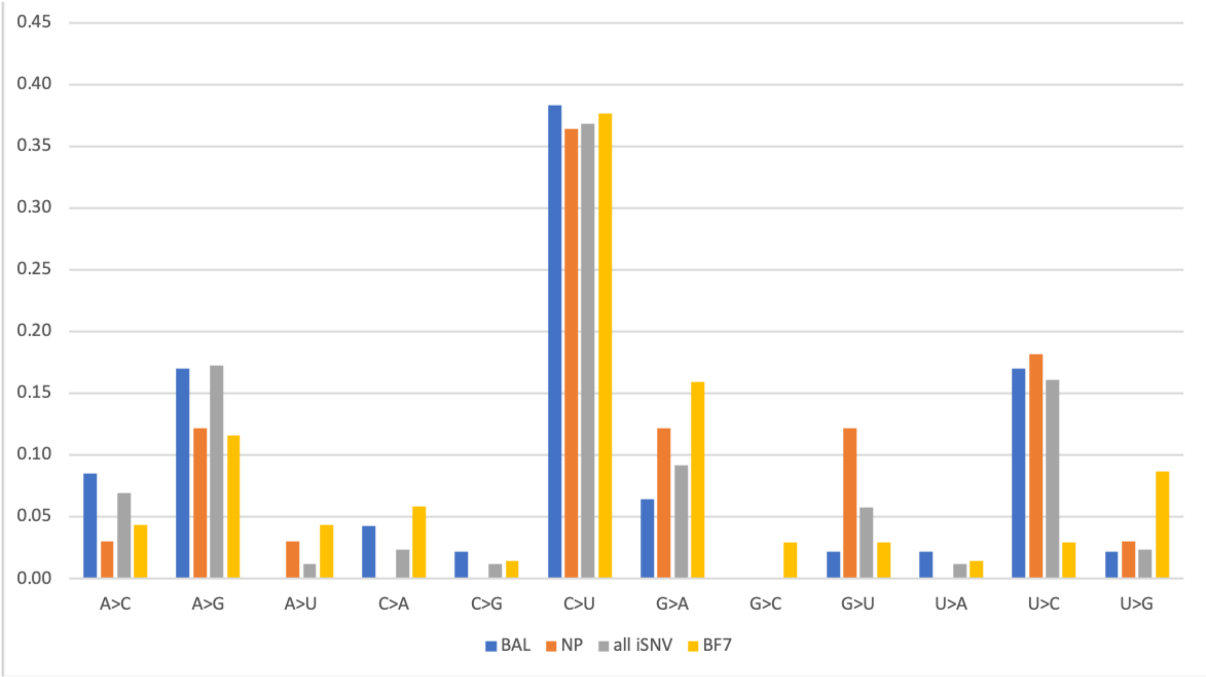

Supplementary Figure S5. Mutation spectra of iSNVs in BAL, NP and all iSNVs, as well as SNVs in ancestral BF.7 lineage.

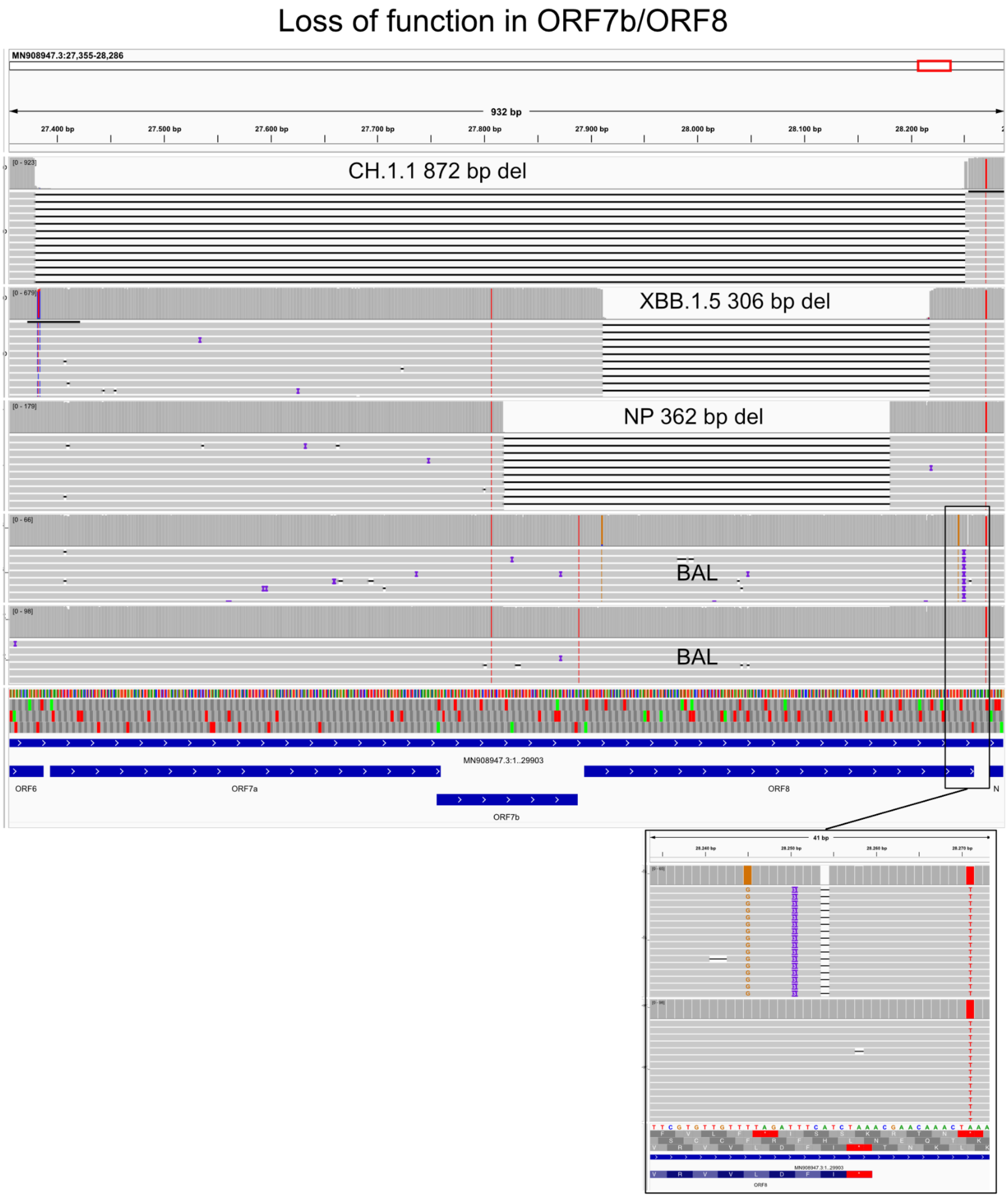

Supplementary Figure S6. Long-range PCR across to ORF7a/b/8 sequenced with nanopore. Two examples of large deletions in circulating lineages (CH.1.1 and XBB.1.5) were observed during routine SARS-CoV-2 genomic surveillance at the University of Liège (Belgium) in 2023. The deletion seen in the NP sample is shown below these. The last two panels show the reads from the BAL separated based on whether they carry the ORF8 frameshift or the wild-type sequence.

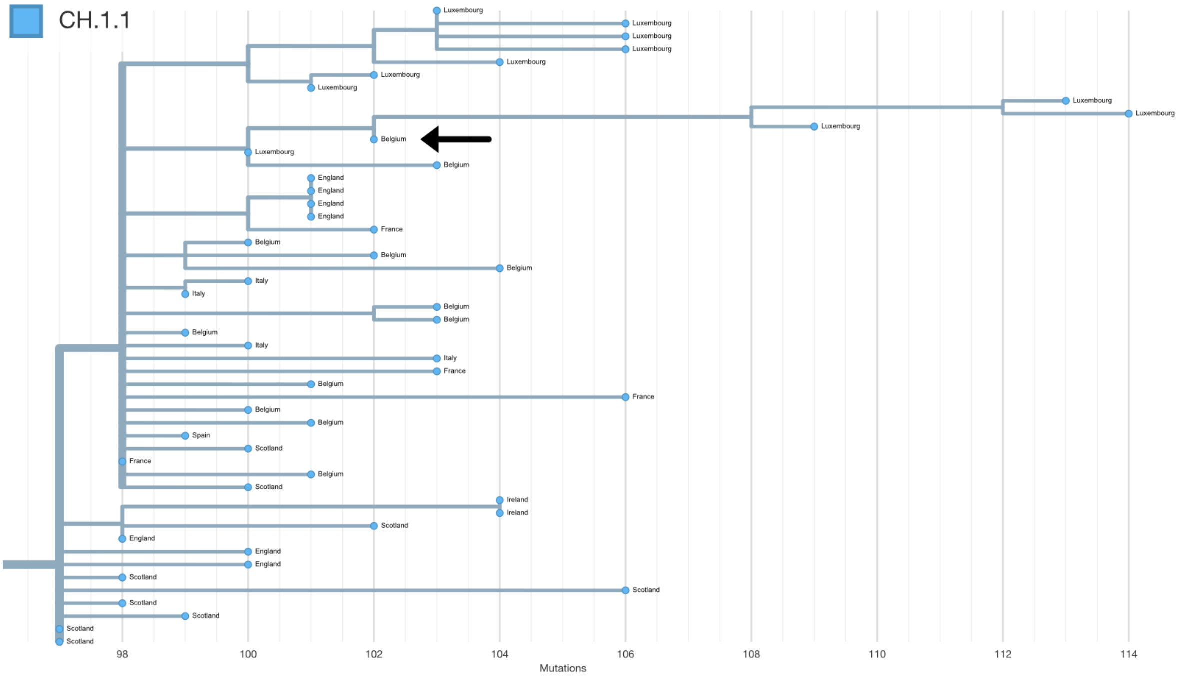

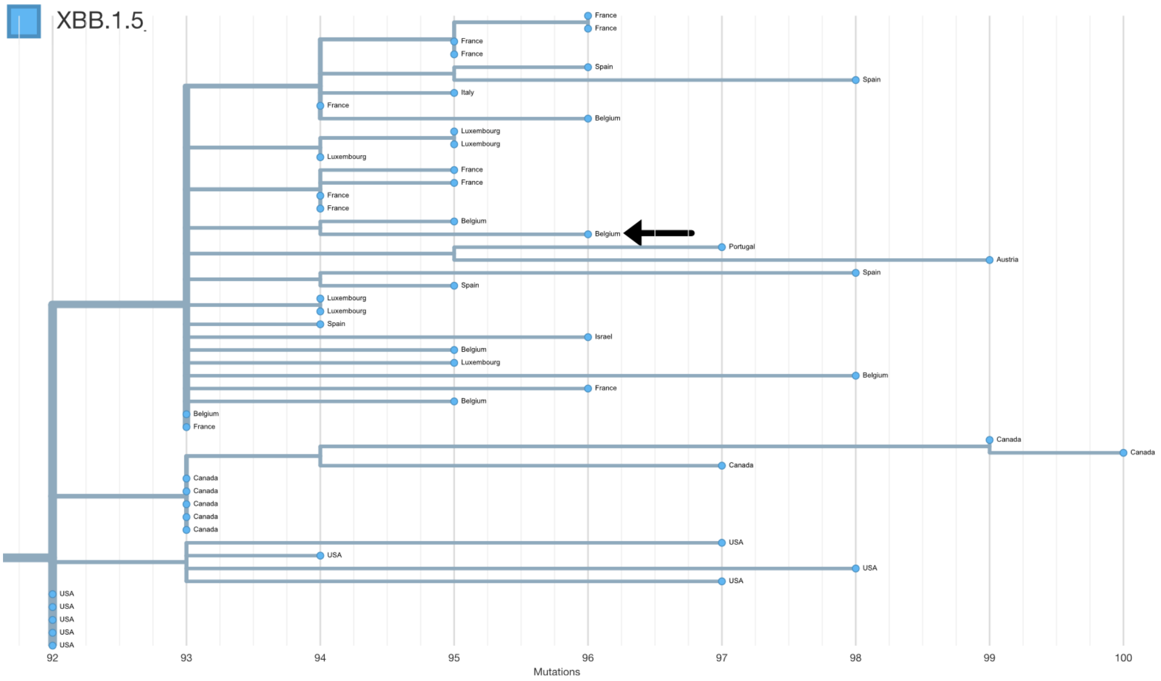

Supplementary Figure S7. Neighbouring samples in the global phylogenetic tree generated by UShER for the CH.1.1 sample with ORF7a/b/8 deletion and the XBB.1.5. sample with ORF8 deletion Both are indicated by an arrow.
